# Barriers to, and enablers of, medication taking among Chinese adults living with type 2 diabetes mellitus in Australia: a qualitative study

**DOI:** 10.1007/s11096-025-01944-w

**Published:** 2025-06-24

**Authors:** Olumuyiwa Omonaiye, Alemayehu Mekonnen, Christopher Gilfillan, Rosemary Wong, Elizabeth Holmes-Truscott, Elizabeth Manias, Bodil Rasmussen, Kevin Mc Namara, Jerry Lai, Louise Huang, Julie Considine

**Affiliations:** 1 School of Nursing and Midwifery and Centre for Quality and Patient Safety Research in the Institute for Health Transformation, Deakin University, Geelong, 1 Gheringhap St, Geelong, VIC 3220 Australia; 2 Deakin University Centre for Quality and Patient Safety Research–Eastern Health Partnership, Eastern Health, Box Hill, VIC Australia; 3 Eastern Health Clinical School, Monash University, Box Hill, VIC Australia; 4 School of Psychology, Deakin University, Geelong, Australia; 5 The Australian Centre for Behavioural Research in Diabetes (ACBRD), Diabetes Victoria, Carlton, Australia; 6 Institute for Health Transformation, Faculty of Health, Deakin University, Geelong, VIC Australia; 7 School of Nursing and Midwifery, Monash University, Clayton, VIC Australia; 8 Department of Public Health, Faculty of Health and Medical Sciences, University of Copenhagen, 2200 Copenhagen, Denmark; 9 Faculty of Health Sciences, University of Southern Denmark, 5230 Odense, Denmark; 10 Deakin Rural Health, Deakin University, Geelong, VIC Australia

**Keywords:** Chinese, Drug therapy, Medication-taking, Medication safety, TDF, Theoretical domains framework

## Abstract

**Background:**

Diabetes prevalence among individuals of Chinese background in Australia is about three times higher than the general population.

**Aim:**

The aim of this study was to identify specific barriers and enablers of medication-taking among adults of Chinese background living with type-2 diabetes mellitus (T2DM) in Australia.

**Method:**

Qualitative semi-structured interviews were conducted with: (1) adults (18 + years) with T2DM of Chinese heritage currently prescribed diabetes medications; (2) health professionals involved in the care of people with T2DM of Chinese heritage. Participants were recruited from a national registry and single specialist clinic. Interviews were audio-recorded, transcribed verbatim, and analyzed using deductive content analysis based on the 14 domains of the Theoretical Domains Framework (TDF). The most relevant TDF domains were identified based on frequency counts across cohorts.

**Results:**

A total of 25 people living with T2DM (60% male; age range: 31–72), and 11 healthcare professionals (5 endocrinologists; 5 pharmacists; 1 credentialled diabetes educator; 10 of Chinese background) participated. Barriers and enablers influencing medication-taking behaviors were identified across 13/14 TDF domains (except for ‘Reinforcement’). Relevant domains most frequently identified were: Social Influences; Environmental Context and Resources; Beliefs About Consequences, and Behavioral Regulation.

**Conclusion:**

Drawing on the TDF, potential targets for future interventions aimed at improving the uptake and maintenance of medication-taking behavior among adults with T2DM of Chinese heritage were identified. These data may support the development of culturally relevant and theoretically informed healthcare professional strategies and diabetes education programs, and ultimately, improve health outcomes for individuals with T2DM.

**Supplementary Information:**

The online version contains supplementary material available at 10.1007/s11096-025-01944-w.

## Impact statements

- The strong influence of family and social support networks was identified as a crucial enabler of medication-taking among people with T2DM, highlighting the importance of involving social supports in diabetes care strategies.
- Frequent out-of-stock issues and the financial burden of medications, particularly for those not covered by government benefits, were significant barriers, suggesting the need for improved access to essential diabetes medications in pharmacy and clinical practice.
- The intersection of cultural beliefs, such as the preference for Chinese herbal medicines over Western prescriptions, and social stigma associated with public medication-taking, underlines the need for culturally sensitive approaches in diabetes education and care.
- The therapeutic relationship with healthcare providers played a central role in medication-taking, emphasizing the importance of trust and ongoing support from healthcare professionals in managing chronic conditions like T2DM.
- People with T2DM utilize various behavioral regulation techniques, such as routines and reminders, to manage their medication schedules, indicating that reinforcing these strategies could be beneficial in clinical practice to improve medication-taking and health outcomes.

## Introduction

Type 2 diabetes mellitus (T2DM) is a significant public health concern [1]. T2DM is increasing across diverse populations globally [2, 3], including Chinese people living in Australia [4]. Australia has experienced a surge in diabetes, with T2DM being the most prevalent form, affecting approximately 85–90% of diagnosed cases [5]. People of Chinese heritage living in Western countries, including Australia, face a significantly elevated risk of developing T2DM compared to individuals of Caucasian origin, with a risk that is at least 60% higher [6]. In Australia specifically, individuals of Chinese background have a diabetes prevalence approximately three times higher than that of the general population [4].

Effective pharmacological treatments exist for the management of T2DM, yet the prescription, uptake, and maintenance of medication remain significant challenges in achieving optimal health outcomes and preventing long-term diabetes-related complications [7]. Vast international research offers insights into the complexity, experiences, and predictors of medication-taking among adults with T2DM overall. [8, 9] However, there remains a need to understand localized experiences of, and support needs for, medication-taking behaviors to inform culturally relevant healthcare provision.

Quantitative research conducted in China identified medication literacy, self-efficacy, and social support as significant factors influencing medication-taking among people with T2DM [10, 11]. Studies in western countries with sizable Chinese communities (e.g. Australia, Canada, United States), have identified suboptimal T2DM medication-taking among individuals of Chinese descent [12, 13] and highlight cultural factors shaping medication-taking behaviors [12, 14]. Specifically, the only known published study regarding T2DM medication-taking among people of Chinese background in Australia showed strong beliefs in traditional Chinese medicine resulted in suboptimal medication-taking and cultural beliefs, social norms, and health literacy significantly influenced medication-taking patterns [14]. This study and the previously mentioned research both explore factors influencing diabetes self-management among people of Chinese background in Australia, with a specific focus on medication-taking. While the previously cited study [14] provided a quantitative overview of self-management behaviors, highlighting cultural influences, our study takes a qualitative design—using a theory-driven approach. This is because theory-based approaches can effectively explain behavior and offer a clear rationale and direction for developing strategies. Through semi-structured interviews with individuals with T2DM of Chinese background and healthcare professionals, we aimed to identify specific, actionable barriers and enablers to medication-taking using the Theoretical Domains Framework (TDF), offering a strong theoretical foundation for future interventions. Language barriers and a lack of culturally sensitive healthcare services can exacerbate suboptimal medication-taking in culturally diverse populations in Australia [15]. Medication-taking among people of Chinese background with diabetes is a critical yet under-researched area. This gap highlights the need for targeted research to understand the unique challenges faced by people of Chinese background in Australia in optimizing diabetes medication use. Understanding T2DM medication-taking behaviors in this population requires a multifaceted approach that considers cultural, social, and environmental factors to inform targeted interventions and improve outcomes.

Using a behavior change lens, the TDF underpinned this study as the conceptual framework [16]. The TDF is a comprehensive tool to identify barriers and enablers influencing behavior, enabling evidence-based strategies [16]. While many theories focus on individual beliefs and motivations [17–19], the TDF also considers social and environmental influences—making it suitable for this study. It has been widely applied in health behavior research, including in chronic conditions such as T2DM [20, 21].

### Aim

The aim of the study was to identify specific barriers and enablers of medication-taking among adults of Chinese background living with T2DM in Australia.

### Ethics approval

The study was approved by the Human Research and Ethics Committees at Eastern Health [ approval number: LR23-016–94795] and Deakin University Deakin University Human Research Ethics Committee [approval number: 2023–140]. Informed consent was obtained from all individual participants included in the study.

## Method

### Study design

This study employed a qualitative research approach. Data were collected using semi-structured interviews with adults with diabetes and health professionals involved in the care of people with diabetes. This study is reported according to the consolidated criteria for reporting qualitative research (COREQ) [22]. The study was conducted between July – December 2023 by a multi-disciplinary research team (with expertise in diabetes care and treatment, medication-taking behavior, patient safety and health psychology).

The conceptual framework for the study was the TDF [16]. The 14 domains of the TDF informed the development of the project aim of this study and guided data collection (i.e., interview questions), analyses and interpretation. Thus, the TDF was used to explain why and under what circumstances diabetes medication-taking occurs.

### Participants and recruitment

*Participants with T2DM:* Convenience sampling was used to recruit people aged 18 years or older, of Chinese heritage (individuals or whose parents were born in regions such as China, Hong Kong, Taiwan, Malaysia, Singapore, Mauritius, Timor, among others residing in Australia) who were prescribed medication for T2DM, including oral hypoglycaemic agents, insulin regimens, non-insulin injectable medication, or any other medication used in the treatment of diabetes, for a minimum duration of one month. Convenience sampling refers to the selection of participants based on their availability, accessibility, or willingness to take part, which can lead to selection bias. People with T2DM were recruited through two methods: i), by direct invitation to those receiving care at a specialist diabetes clinic at Eastern Health and ii) accessed from a National Diabetes Services Scheme (NDSS) database, an initiative by the Australian Government managed by Diabetes Australia. Registrants on the NDSS database had the option to indicate their willingness to be contacted for future research endeavours, facilitating support for diabetes research in Australia. Those who indicated interest in interviews were contacted via phone (or email if phone number was not provided). As a token of appreciation, AUD$50 e-vouchers were offered. The study aimed to interview 20─30 people with T2DM, with the final count depending on the occurrence of repeated patterns of findings. The sample range was determined based on evidence from the literature [23, 24].

*Health professional participants:* A convenience sample of healthcare professionals who provided care to individuals with T2DM of Chinese heritage at Eastern Health hospitals/and or clinics for at least three months were invited via email. Endocrinologists, credentialled diabetes care and education specialists, and pharmacists received emails from the lead investigator, with further support from department heads who circulated the invitation in their own professional circles. Interested professionals were invited to contact the lead investigator for more information and to provide informed consent. The research team aimed to conduct five interviews for each of the above-mentioned health professional groups [25]. The number of participants required for the study was dependent on the repeated patterns of information obtained from the individuals during the interview.

### Data collection

A total of 25 individuals living with type 2 diabetes mellitus (T2DM) were interviewed. The interviews lasted an average of 18 min, ranging from 8 to 43 min. Nine participants were recruited through the NDSS database. These interviews were conducted either by phone (n = 3) or via Zoom (n = 6). Of the nine interviews, seven were conducted in English (n = 7). Interpreter services were utilized for participants who were not proficient in English — one interview was conducted in Mandarin and one in Cantonese. They were led by one of the authors (O.O.), a male public health researcher with a PhD with extensive experience in conducting qualitative interviews with individuals from culturally and linguistically diverse backgrounds.

An additional 16 consenting participants were recruited from a specialist diabetes clinic at Eastern Health by a dedicated research assistant (L.H.), a female registered nurse of Chinese descent, experienced in conducting research with people of Chinese background and fluent in both traditional and simplified Chinese. These interviews were conducted either face-to-face in a clinic room (n = 9) or by telephone (n = 7). Of these, seven interviews were conducted in Chinese and nine in English.

Demographic information was collected from people with T2DM and health professionals before the start of the interviews and interviews were digitally recorded. The interview guide for people with T2DM and health professionals are included as appendices (Supplementary material S1).

### Analyses

English audio-recordings were transcribed verbatim by a professional transcribing service, while in-language interviews were transcribed by LH. NVivo 2020 Software (QSR International Pty Ltd) was used for data management. Since transcription was undertaken verbatim, there were some grammatical issues noted but these were left unchanged to preserve the accuracy and context of the communication. Interview transcripts were analyzed using directed content analysis [26], applying the 14 TDF domains as a coding framework in a deductive manner [16]. During the coding phase of data analysis, two research team members (OO and AM) independently conducted the coding, enabling discrepancies to be resolved through discussion, review of original transcripts, and regular engagement with other team members (CG, RW, EHT, EM, BR, KMN, JL, LH, JC). These consultations provided a platform for discussion, facilitating the refinement and synthesis of textual content into the final behavioral domains. Contextualized brief statements reflecting the determinants of medication-taking behavior in people with T2DM were identified across the interviews and categorized and mapped into the TDF domains. The theoretical domains judged to be relevant were identified by considering the frequencies of the beliefs reported, the presence of conflicting beliefs, and evidence of strong beliefs that may influence the behavior of medication-taking across and within participants cohorts (i.e. participants with T2DM and health professionals) [27]. In assessing domain relevance, all these factors were considered concurrently.

### Trustworthiness of the data and interpretation

Various strategies and procedures were integrated into the study to ensure the credibility, dependability, confirmability, and transferability of the findings [28]. Regular peer briefings were conducted through email updates to keep the project team informed about the ongoing research activities, including recruitment and data collection. The primary research setting for recruiting individuals with T2DM was the specialist diabetes clinic, where the research assistant (L.H) had sustained involvement over several months, enabling familiarity with the study’s participants. The two investigators (O.O, L.H.) engaged in recruitment and data collection possessed the necessary knowledge, research skills from prior experience, training and education, and cultural competence to effectively perform their roles. A detailed study protocol was prepared and followed throughout the study, and a detailed record of the data collection process was maintained. Two researchers (O.O, A.M) coded data independently into theoretical domains following a mutually agreed coding guideline to increase the consistency of coding. We applied several triangulation techniques (data source (from both people with T2DM and health professionals), investigators and theoretical). Furthermore, all interviews were conducted with the support of professionally trained interpreters. To enhance accuracy, the interpreters were briefed prior to the interviews on the study’s aims, key terminology, and the importance of maintaining both linguistic and cultural nuances in translation. The bilingual team members participated both in data collection and analysis. Their linguistic and cultural expertise played an important role in verifying and interpreting meaning within the translated responses, thus guarding against potential misinterpretations of culturally embedded expressions.

## Results

### Participant characteristics

The average age of people with T2DM was 54 (range: 31─72 years), 60% were males and 60% were born in China. No new themes were coded among people with T2DM between the 23rd and the 25th interview indicating data saturation at the 23rd interview. Most participants with T2DM took oral diabetes medication only (n = 16) and had been prescribed diabetes medications for more than five years. All but one person with T2DM lived within one hour of their hospital and most (n = 19) reported that they had a support person who encouraged them to take their medications as prescribed. Table 1 presents further information about participants with T2DM. Of the healthcare professionals, five each were endocrinologists and pharmacists, all with a Chinese background and one was a credentialled diabetes care and education specialist (not reporting Chinese heritage).

**Table 1:** Characteristics of people with T2DM

Characteristics	Value
Age, mean (range)	54.44 (31─72)
Gender (male), n (%)	15 (60)
Country of birth	
China	15 (60)
Hongkong	5 (20)
Australia	3 (12)
Malaysia	2 (8)
Language spoken at home, n (%)	
Mandarin	8 (32)
Cantonese	4 (16)
English	3 (12)
English/Cantonese	3 (12)
English/Mandarin	3 (12)
Others*	3 (12)
Education, n (%)	
Primary	1(4)
Secondary	4 (16)
Tertiary	20 (80)
Marital status, n (%)	
Married	16 (64)
Single	4 (16)
Widowed	3 (12)
Separated/divorced	2 (8)
Travel time to hospital, n (%)	
< 1 h	24 (96)
> 1 h	1 (4)
Type of diabetic medication, n (%)	
Oral only	16 (64)
Oral and Insulin injection	9 (36)
Duration on diabetic medication, n (%)	
More than five years	17 (68)
Less than five years	8 (32)
Received regular encouragement to take medicines, n (%)	
Yes	19 (76)
No	6 (24)

*Others (Mandarin/Malaysian: 1; English/Mandarin/Cantonese:1; Mandarin/Cantonese: 1)

### Barriers to, and enablers of, medication-taking behavior

**Fig. 1:**
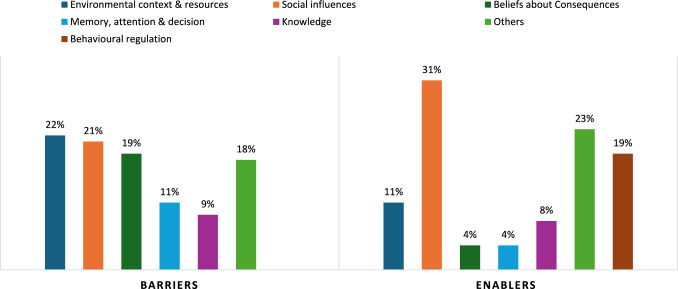
Top five predominant domains identified

In examining factors influencing medication-taking behavior, barriers and enablers were identified in 13 of the 14 TDF domains; no influences were identified within Reinforcement. The TDF domains are ranked based on relevance in Table 2. The most common barriers were Social Influences, Environmental Context and Resources, and Beliefs About Consequences, while the most common enablers were Social Influences, Behavioral Regulation, and Environmental Context and Resources. Notably, Social Influences and Environmental Context and Resources were both major barriers and enablers. Table 2 presents sub-category themes along with the number of barriers and enablers identified, while supplementary material 2 provide a comprehensive list with illustrative quotes. A narrative summary of the three most common barriers and enablers is found below. Figure 1 presents the top five predominant domains identified.

**Table 2:** Barriers and enablers to medication-taking behaviour by TDF domains, subcategory themes and research participant group with coding frequencies

Domains	Barriers				Enablers			
	Subcategory theme	T2DM	HCPs	Total	Subcategory theme	T2DM	HCPs	Total
Knowledge	PWT2DM lack knowledge about DM, medications, and management	10	5	15	PWT2DM has good understanding of DM, medications, and management	10		10
	Support person lack of knowledge about DM	1		1	Education	1	1	2
	HCPs lack of knowledge on Chinese medicines	2		2				
Skills	Communication and language barrier	2	2	4	Insulin self-administration	2		2
	Suboptimal injection technique		1	1	Blood glucose monitoring and insulin dose adjustment	3		3
					Good Communication skills		1	1
Social/professional role and identity	Cultural variation of illness identity	2	2	4	Health consciousness		1	1
	DM stigma		1	1	Non-discrimination	1		1
	Cultural dependency on young		1	1	Pragmatism		1	1
Beliefs about capabilities	Lack of competence in self-administration	1	1	2	Regular follow-up		1	1
	Lack of confidence in HCPs ability of DM management	1		1	Confidence in DM management	4	0	4
	Refusal to accept a lifelong management	0	2	2				
Optimism	Uncertainty about medications impact	1		1	High confidence in medicines benefit	3		3
					Non-invasive future new technology	1		1
Beliefs about Consequences	Side effects of medicines	9	5	14	Strong beliefs about medication benefit	5	1	6
	Chinese vs Western Medicine dilemma		7	7				
	Medication burden	1	3	4				
	Preferences for oral medications to injections		3	3				
	Underestimation of medications benefit and high confidence in diet	2	1	3				
	Less emphasise on prevention and regular check-up	2		2				
Intentions	Intention to stop or skip medications	1		1	Willingness to intensify or adjust treatment as needed	2	0	2
					High engagement with DM routines	3	0	3
Goals					Wish for live longer	1		1
Memory, attention and decision processes	Forgetfulness to take medications	7	2	9	Webster-paking and other medication packages	3	2	5
	Mental burden	1	1	2	Carbohydrate consciousness	1	0	1
	Unsubstantiated personal decision	3	0	3				
	Busy and urgent work schedule	2	0	2				
	Lack of routine	2	0	2				
	Lack of reminder support	1	0	1				
Environmental context and resources	Financial constraints	1	5	6	Brochures and resources in other languages	0	4	4
	Staff shortage and time constraints	0	5	5	Chinese speaking HCPs and interpreters	0	4	4
	Differences in work condition and culture	2	3	5	Detailed medication history taking		2	2
	Challenges in sustaining access to essential medications	2	2	4	Extended pharmacy and community services	1	2	3
	Access to many food options; high carbohydrate diet	3	1	4	Availability of MDT team and more HCPs		1	1
	Language barrier	1	2	3	Availability of technology		1	1
	Medication and dose regimen changes	1	2	3				
	Lack of material resources and information	3	2	5				
	Systemic issues (e.g. limited Chinese interpreters, longer appointment, etc.)		3	3				
Social influences	Lack of family or peer support	5	0	5	Trust and follow HCPs instruction	12	3	15
	Preferences for Chinese or alternative medications to western medications	1	2	3				
	Refusal to accept recommendations	1	1	2	Family and peer support	8	5	13
	Negative feedback about DM medications from family and peers	3	2	5	Joining support groups	2	1	3
	More reliance on Chinese medicine and doctors	1	6	7	Building positive therapeutic relationship	0	2	2
	Stigma and fear of DM label	0	3	3	Liaison with HCP	0	1	1
	lack of information sharing with family and peers	1	1	2	Sharing information with family and peers	4	1	5
	Feeling shame taking medications in public	6	1	7	Self-empowerment		1	1
	Old beliefs about DM		1	1	Home medication-taking	2	0	2
	Lack of trust of HCPs	1		1				
Emotion	Anxious about regular medication-taking	4	1	5	Taking life/medication for granted	3		3
	Worry about long-term side effects of medications	2	0	2				
	Worry about taking many medications together	1	0	1				
	Worry about long-term complications of DM	1	1	2				
	Feeling tired	1	0	1				
Behavioral regulation	Challenge in finding the right routine	1		1	Prevention measures to medications missing and side effects	9	1	10
					Positive medications outcome		2	2
					Prevention measures to DM and its complications	1	0	1
					Adapt to routines	7	0	7
					Apps and clock alarms	3	0	3
					Book recording	2	0	2
					Keep medications closer to your sight	1	0	1

DM: diabetes mellitus; HCPs: healthcare providers; MDT: Multidisciplinary team; PW2DM: People with type 2 diabetes mellitus

The numbers in the table represent coding frequencies, indicating the number of times each (sub)theme was identified within the responses from two research participants groups—people with T2DM and healthcare providers. The 'Total' column sums the coding frequencies for each subcategory theme

### Barriers to medication-taking behavior

#### Domain: Environmental Context and Resources

This was the most common barrier. Participants with T2DM struggled with medication shortages, causing frustration when visiting multiple pharmacies. As one participant remarked:

"Generally, it’s okay, but the injection is always running out of stock. I have to visit different chemists to buy it. It’s hard to keep it in stock." PW2DM_6.

Healthcare professionals echoed these concerns:

"I know patients try to adhere to medications, but if they can’t get the stock, they end up giving up... the most popular one still ongoing is Ozempic." Pharmacist_002.

For those without access to the Government Pharmaceutical Benefit Scheme, financial burdens were significant:

"...it’s financially challenging. My pension doesn’t cover all the costs, and I have to use my Chinese pension to buy medication here. I am not qualified for benefits here yet." PW2DM_15.

#### Domain: Social Influences

Social influences also shaped medication-taking. Participants and professionals highlighted the impact of traditional Chinese medicine. Pharmacist_005 noted,

"I think... definitely Chinese medication has a big influence on how they manage their diabetes... because I come from a Chinese background myself."

Another professional added:

"I have some patients who either inquired or tried Chinese medications to help with diabetes control." Doctor_001.

Despite living in Australia, some participants consulted Chinese doctors overseas:

"I think the metformin sometimes... not control my sugar... but the Chinese doctor is saying you can try some herbal and plus exercise... So, I just taking some herbal and plus doing the regular exercise, I think that works for me." PW2DM_24.

Healthcare professionals often saw patients combining herbal and prescribed medications. Doctor_004 explained:

"There have been cases where patients went overseas, were introduced to a local remedy or herbal treatment, stopped their Western medications, tried that out, and then came back. Obviously, it’s affected their management."

To navigate this, professionals maintained a balanced approach. Doctor_003 stated:

"I still respect Chinese medicine... I don’t know its interaction with prescribed medicine because there’s no data. If they prefer to continue Chinese medicine, please don’t stop the prescribed medication and watch for side effects."

Cultural barriers also influenced medication-taking, with participants feeling uncomfortable taking medication publicly:

"Sometimes when I go out with my friends, I don’t want to take Metformin in front of them because I don’t want to explain too much." PW2DM_22.

"I’m not comfortable taking medication in public. I’m not accustomed to sharing my health conditions; it’s not necessary." PW2DM_5.

Healthcare professionals observed similar issues:

"I have patients who must inject three to four times daily. They tell me, ‘Doctor, I’m sorry, I can’t do so many injections at work. It’s embarrassing.’ " Doctor_002.

#### Domain: Belief About Consequences

This was the third most common barrier. Participants shared concerns about illness, medication side effects, and glycaemic management:

"When you’re quite sick and not eating, Metformin suppresses your appetite even more, making it difficult to get basic energy." PW2DM_19.

Some feared long-term effects:

"In the long run, it’s [medication] going to be damaging to the rest of my organs." PW2DM_1.

Healthcare professionals confirmed these fears:

"The most common factor lowering compliance is side effects." Doctor_003.

Some participants stopped medication entirely:

"Consequently, I ceased taking my prescribed oral tablets, believing I could manage this health condition without medication." PW2DM_14.

### Enablers of medication-taking behavior

#### Domain: Social influences

Despite being a barrier, social influences also acted as an enabler. Family support played a key role, with reminders and assistance:

"They [wife and children] would remind me… Half the time I would say I remember." PW2DM_19.

"If I couldn’t go buy the medication, they would get my prescription and help me." PW2DM_22.

Understanding from family and friends motivated medication-taking:

"So all my family and friends are fully aware of that…, everyone embraces the situation... diabetes itself, tablet, no problem in our family." PW2DM_18.

Trust in healthcare professionals also influenced medication-taking:

"I listen to doctors. I trust them. I take my medications on time." PW2DM_5.

#### Domain: Behavioral Regulation

Behavioral Regulation was the second most common enabler. Given that T2DM requires lifelong management, participants relied on routines, reminders, and action planning:

"I keep them [my medications] on my bedside table, along with my other morning medications, so I don’t forget." PW2DM_15.

Some developed structured medication plans:

"I’ve got a cosmetic bag with all my medications. In the morning, I leave it on my bench... I know which one to take at breakfast, lunch, and dinner." PW2DM_1.

Digital reminders also helped:

"I use the Apple iPhone medication app and set a reminder." PW2DM_18.

However, some struggled with consistency:

"I always struggled with taking medication regularly. I bought a pill box, but that didn’t help because I would forget about the box itself." PW2DM_19.

#### Domain: Environmental Context and Resources

Despite being the most common barrier, Environmental Context and Resources also served as an enabler. Healthcare professional support improved medication-taking:

"My pharmacist takes charge of my medication and delivers it to my place. I don’t need to pay either. He helps me restock every time." PW2DM_14.

Follow-up care also played a role:

"Two days after discharge, a pharmacist calls to check in: ‘We’re calling from Eastern Health to follow up. Were there any questions about medicine?’ " Pharmacist_001.

## Discussion

### Statement of key findings

People with T2DM and healthcare professionals, identified a diverse array of barriers and enablers, across 13 out of 14 TDF domains, that could influence medication-taking behaviors among adults with T2DM living in Australian of Chinese heritage. The most common barriers were *Environmental Context and Resources, Social Influences,* and *Beliefs About Consequences, with consistency across cohorts.* The most common enablers were *Social Influences, Behavioral Regulation* and *Environmental Context and Resources.* It is important to note that *Environmental Context and Resources* and *Social Influences* were both powerful barriers and enablers*.* These barriers and enablers highlight potential targets for future culturally relevant interventions aimed at improving medication-taking behavior.

### Interpretation

#### Barriers

The most common barrier was Environmental Context and Resources. Participants with T2DM often faced medication access issues due to stock shortages, causing frustration and hindering medication-taking. This highlights the need to address supply chain challenges, aligning with WHO policy [29] on healthcare infrastructure and access.

While supply chain challenges related to diabetes medications like Ozempic (semaglutide) are not unique to individuals of Chinese descent with T2DM in Australia, our findings highlight additional barriers influenced by cultural beliefs and social factors (Social Influences). The preference for Chinese herbal medicine alongside Western medications reflects a complex interplay between personal beliefs and social expectations [30]. However, this integration presents challenges for healthcare professionals seeking to optimize T2DM management [31], as some participants expressed limited understanding of disease management and available treatments. The concurrent use of traditional and Western medicine is well-documented beyond our study setting [32], yet healthcare professionals’ limited knowledge of traditional Chinese medicine may hinder informed decision-making when treating patients who favor herbal remedies [31].

These findings align with previous studies advocating for culturally tailored diabetes education to address knowledge gaps and enhance both self-management [33, 34] and medication-taking practices [35]. A recent systematic review reported that culturally tailored, in-person Diabetes Self-Management Education (DSME) in low- and middle-income countries, particularly sub-Saharan Africa, improved diabetes outcomes, including reductions in glycated hemoglobin [36]. Similarly, a U.S.-based community DSME program for Korean immigrants showed significant improvements in hemoglobin A1c, waist circumference, and HDL cholesterol over a three-month period [37]. Participants’ beliefs about medication consequences—such as concerns about side effects and long-term organ damage— posed additional barriers to medication-taking. Addressing these concerns through education and shared decision-making can empower patients to make informed choices about their treatment [38].

#### Enablers

Despite the barriers, social support—especially from immediate family—served as a major enabler of medication-taking. Participants cited reminders and help with refills as key supports, and acceptance by family and friends created a positive environment for diabetes care. Among Chinese communities, family plays a central role in chronic disease management, including T2DM [39]. These findings reinforce the importance of involving social networks in diabetes education and care. Research from China similarly underlines the impact of social support on medication-taking among people with T2DM [10]. Establishing consistent medication routines (Behavioral Regulation) also emerged as essential for medication-taking. Strategies such as medication reminders and organizational tools facilitate medication-taking by promoting consistency and reducing forgetfulness [40, 41]. However, challenges in establishing effective routines highlight the need for tailored support and interventions. Healthcare professionals should co-create personalized medication plans and incorporate behavioral support into diabetes care to optimize medication-taking and overall health outcomes.

Healthcare services and individualized medication management were additional enablers. Participants appreciated interventions such as home delivery of medications and follow-up calls, which improved access and offered ongoing support. Personalized care and practical solutions can help mitigate logistical challenges, supporting long-term medication-taking and better outcomes [38].

### Policy recommendations

To improve medication-taking, policies should enhance accessibility through subsidy programs and streamlined pharmacy distribution. Culturally tailored health education should address concerns about combining Western and Chinese medications. Community awareness campaigns can reduce social stigma. Digital reminders and pharmacist-led follow-ups should be integrated into diabetes management. To ensure cultural acceptability among older Chinese Australians, reminders should use relevant language, symbols, and imagery—such as traditional idioms or visuals that resonate culturally. Strengthening connections between interventions and specific TDF domains would enhance theoretical coherence. For example, to address culturally influenced beliefs about medication efficacy and harm (beliefs about consequences), we propose educational materials that integrate traditional Chinese health beliefs with biomedical views. Involving respected community health figures or bilingual pharmacists can boost cultural relevance and credibility.

### Strengths and weaknesses

The strength of this study lies in its use of the TDF to analyse the study data, which facilitates the identification of potential interventions grounded in theory. Another strength of the study is the inclusion of perspectives from both people with T2DM and health professionals, most of whom share the same cultural background as the participants. This approach enriched the data collected, providing a more comprehensive understanding of the study topic. However, despite addressing a significant knowledge gap the study has several limitations. The rich perspectives and responses obtained from small, self-selected samples may not fully represent the broader Chinese population in Australia, thereby limiting the transferability of the findings. Because of the broad definition of "Chinese heritage" in this study, which is inclusive, covering various regions and countries, we acknowledge that this broad categorization may mask potential subcultural or linguistic differences. This categorization may also limit the transferability of the study findings. This limitation also applies to the group of participating health professionals. Additionally, social desirability and recall bias during interviews could have influenced the data collection process and should be taken into consideration when interpreting the results.

### Further research

Future research should examine the influence of cultural beliefs and social factors on medication-taking behaviors among diverse populations, including the integration of traditional and modern healthcare practices. Research could focus on understanding the reasons behind individuals’ preferences for alternative treatments, exploring culturally tailored interventions such as testing the effectiveness of culturally tailored medication-taking interventions or the implementation of digital health tools that are adapted for specific populations to promote medication-taking, and assessing the impact of cultural competence training for healthcare professionals Future research could focus on developing culturally tailored, multifaceted intervention strategies that address both enablers and barriers to medication-taking, ensuring inclusivity and adaptability for culturally and linguistically diverse populations in Australia.

### Conclusion

Addressing the multifaceted challenges to medication-taking in people with T2DM requires a comprehensive, person-cantered approach. By considering environmental, social, emotional, belief-related, and behavioral factors, healthcare professionals can enhance motivations for, uptake and maintenance of medication-taking, and ultimately improve health outcomes for individuals with T2DM.

## Supplementary Information

Below is the link to the electronic supplementary material.

Supplementary file1 (DOCX 20 KB)

Supplementary file2 (DOCX 30 KB)
