# Supplementary material for: Barriers to, and enablers of, medication taking among Chinese adults living with type 2 diabetes mellitus in Australia: a qualitative study
Source: Int J Clin Pharm. 2025 Jun 24;47(6):1748–59. doi: 10.1007/s11096-025-01944-w (PMC12630298; doi:10.1007/s11096-025-01944-w)
Supplement: Supplementary file 1 — Supplementary file1 (DOCX 20 KB) [file 11096_2025_1944_MOESM1_ESM.docx]

**People with T2DM: Interview guide for semi-structured interview**

Investigating diabetes medication taking among individuals of Chinese heritage

Brief Introduction: The purpose of this interview is to understand how you feel about your diabetes and about your diabetes medication taking behaviors. There is no right or wrong answer.

I want to remind you that participation is voluntary. You don’t need to answer questions if you do not want to, and we can stop the interview at any time.

The interview will last no longer than an hour and will be audio recorded.

Do you have any questions before we begin?

Semi structured interview guide and data collection form

Responses will be tape recorded.

1. Can you tell me a bit about yourself? Where do you live / what are your living arrangements/ what kind of work do you do?
2. Can you describe what a typical day would be for you?
3. How is diabetes viewed among the Chinese community?
4. What prescribed medications are you currently taking for your diabetes?
5. Do you know every medicine that you take for your diabetes?
6. Do you know how take all your medications? E.g., injecting insulin> Do you have to depend on someone to help you inject your insulin?
7. How do you feel about taking medications to manage your diabetes?
8. What do you understand about the good things of taking your diabetic medications?
9. What do you think is the most unpleasant things about taking your diabetic medications?
10. Do you have any worry about taking your diabetic medications?
11. How important is it to you to take all of your different medications as prescribed by the GP?
12. When would it be less important to you to take all of your different medications as the GP has prescribed?
13. What do you think the benefits are of taking all of your medications as prescribed?
14. How involved are you in making decisions about your medications, when you see the doctor?
15. What influence do the people in your life, (such as family members, friends, people in the community) have in helping you manage your diabetes? How do they support you to take your medications?
16. Some people miss or skip their medications from time to time. What has been your experience with missing or skipping medications?
17. Do you have any routines or habits that help to make sure you take your medications as prescribed? And apart from forgetting to take your medicines, would you ever decide not to take your medicines as prescribed? What would be the reasons that you would deliberately decide not to take the medicines as prescribed?
18. What things do you do to help you to take your diabetes medications?
19. How comfortable are you with taking your diabetic medications in public, such as at work or during family gatherings?
20. What things in your day-to-day activities affect whether you take your medications as planned?
21. Do you have anything else that you would like to share with me?

**Interview guide and data collection form for semi-structured interview with health professionals**

Responses will be tape recorded.

1. Can you tell me a bit about yourselves and the scope of your work in the diabetes clinic and or what you do in relation to treatment, care and support for people with type 2 diabetes?

2. Can you describe your typical day in terms of the work you do in the diabetes clinic? [Prompt: how effective are you in supporting patient adherence to medicines (and specifically Chinese patients), and why]

3. From your work experience what factors do you think have a positive influence on adherence to diabetes medications among Chinese adults receiving treatment for type 2 diabetes?

4. From your work experience what factors do you think have a negative influence on adherence to diabetes medications among Chinese adults receiving treatment for type 2 diabetes?

5. Are there factors associated with healthcare delivery that promote medicines adherence for patients with diabetes, and particularly Chinese patients? [Are any system or infrastructural and system factors within your hospital/clinic that facilitate adherence among Chinese adults receiving treatment for type 2 diabetes?] **Prompt**: Then prompts: Ask specific factors relating to health professionals (e.g. attitudes/workload), the health service (e.g. inadequate protocols), and the health system determinants (e.g. workforce shortages) etc.

6. Are there factors associated with healthcare delivery that inhibit medicines adherence for patients with diabetes, and particularly Chinese patients? [ Are any system or infrastructural and system factors within your hospital that inhibit adherence among Chinese adults receiving treatment for type 2 diabetes?] **Prompt:** Then prompts: Ask specific factors relating to health professionals (e.g., attitudes/workload), the health service (e.g., inadequate protocols), and the health system determinants (e.g., workforce shortages) etc.
