# Supplementary material for: Barriers to, and enablers of, medication taking among Chinese adults living with type 2 diabetes mellitus in Australia: a qualitative study
Source: Int J Clin Pharm. 2025 Jun 24;47(6):1748–59. doi: 10.1007/s11096-025-01944-w (PMC12630298; doi:10.1007/s11096-025-01944-w)
Supplement: Supplementary file 2 — Supplementary file2 (DOCX 30 KB) [file 11096_2025_1944_MOESM2_ESM.docx]

Illustrative quotes by TDF domain and barriers and enablers

| **Domains** | **Illustrative quotes** | |
| --- | --- | --- |
|  | **Barriers** | **Enablers** |
| **Knowledge** | ‘’I don’t know [ prescribed medications taking], actually.’’ PW2D_3  " I felt like they don't really care about that. In general, they lack awareness of their health." PW2DM_11  “I’ve noticed a lack of information about diabetes, especially regarding diets, in the Chinese version.” PW2D_2  "When they feel normal because moderate elevated high sugar levels, people won’t have any symptoms and they thought, I don’t have symptoms, I’m healthy. Why do I need to be worry? So, this reflected lack of knowledge about diabetes and it’s complications." Doctor_003 | "Realizing my lack of knowledge about diabetes, I felt that diabetes education is necessary" PW2DM_13  "I believe - I'm confident that I have enough knowledge and am really aware of that but I don't think the general public have a very good knowledge about that." PW2DM_20  ‘’So, I do discuss it with him [Doctor] a lot. I have questioned him about whether I need to reduce the tablet, maybe go on one once per day, or do I really need it. But I do rely on his expertise and after all he's a doctor, I'm not.’’ PW2DM_18 |
| **Skills** | ”So, if they don’t know how to use the device, obviously, they’re not going to use it. Therefore, they’re not going to be adherent to that medication.” Pharm02  “… due to language barriers, I haven't attended any support groups. My English level is poor.” PW2DM_13 | “She [wife] accompanies me to pharmacy visits and doctor appointments. However, I administer the injections myself as she's afraid of needles” PW2DM_7 |
| **Social/professional role and identity** | "So I guess this is a little bit special about the Chinese ethnicity with diabetes." Doctor_002  " I think with this population - I suppose with any other ethnic populations - the main concern with them is the understanding of the illness itself. A lot of these patients, unfortunately, fail to understand the importance of controlling diabetes or managing diabetes" Pharmacist_002  As they get older, the culture - specifically in Chinese culture - the younger people tend to do everything for the elderly. Even if they are physically well, mentally well. I feel like there’s a cultural dependence on the younger” Pharm5 | ‘’They are keen to manage their diabetes. I don’t know if it’s particular to Chinese people, but I do find that the Chinese people do tend to be more likely to adhere to guidelines and to intervention and to medication suggestions.’’ DNE_001  I’m thinking like a lot of people got that and the most important thing is that don’t discriminate a patient who has diabetes, it’s just normal people. PW2DM_ 24  "the Chinese people overall will take their health very seriously" DNE_001 |
| **Beliefs about capabilities** | ‘’It’s kind of like – I think they do feel like they’ve failed a bit in terms of their management, they’ve not been able to fix things themselves with whatever they can do when they’ve got to the stage of needing insulin treatment, mmm.’’ Doctor_004  “If BGL [blood glucose level] was high, I will give myself extra dose of insulin. However, when I saw my GP yesterday and he told me this is a wrong way to do. My GP prescribed me another short-acting insulin which can lower my BGL more quickly. “ PW2DM_2 | ‘’Otherwise, I think 90 per cent – it’s all from myself. I don’t need anyone to help me or mention about it or need to help me.’’ PW2DM_4  ‘’Not really. I handle it [medication-taking] independently. ‘’PW2DM_6    ‘’My wife reminds me sometimes, but mostly I manage them myself. I administer the insulin on my own.’’ PW2DM_16  "I guess quite easy and they also give me confidence that it's under control." P20 |
| **Optimism** | ‘’I don't - I'm uncertain about the long-term effects medication can have, because Metformin it’s a tablet, right, I don't know because I'm not trained in chemistry or in pharmacy, right, I don't know what is the composite off this medication and whether any element inside can impact on my body.’’ PW2DM_17 | ’I feel like I take medication, I feel nothing wrong. Everything is normal, so that means I’m not worried about it.’’ PW2DM_4  ‘’Yeah. Look, the motivation is maybe one day I can completely get off the medication. I've also been lately doing a lot of research and listening to health people about gut health medication and all that. I know a lot of them said, yes, you can get away from medication.’’ PW2DM_18  "So, I hope – sometimes I hope there is new technology there will be meters, you don’t need to draw to get the blood right, is that the new technology, do you know that? I’m very interesting to buying or get one, new meter that don’t have to get a needle [laughs] to get the blood every day." PW2DM_24 |
| **Beliefs about Consequences** | ‘’In the long run it’s [ medication] going to be damaging to the rest of my organs in the body, you know.’’ PW2DM_1  “If I'm unsure whether I've taken the medication or not, I just leave it. I worry more about a double dose than missing medication." PW2DM_2    ‘’I'm actually unsure what the side effects, long term side effect could be from those tablets.’’ PW2DM_17  “They don’t undergo body check-ups every year or regularly. They usually seek Chinse medicine therapy when they feel unwell. They also neglect the importance of prevention at earliest stage. It becomes too late to change your diet and adjust your lifestyle once you have been diagnosed diabetes. They regret that they should have done something earlier to prevent its progression” PW2DM_11  "Consequently, I ceased taking my prescribed oral tablets, believing I could manage this health condition without medication.” PW2DM_14  "Medication – so combination tablets – having two drugs in the one tablet, is also – can actually impact significantly on patient adherence as well” Doctor_001  ‘’The most common factors that lower their compliance is the side-effect and the second one would be the cost and third one, I guess, would be how easy the treatment will be. For example, they’re more compliant to tablets compared to injections.’’ Doctor_003 | ‘’Not at all, because like I said I've been taking Jardiamet for so long and I'm happy with it. Like I said, the Diabex before was giving me stomach problem. But now I don't seem to have that sort of problem as much.’’ PW2DM_18  ‘’Also, this helps with suppressed - what's the - supressed appetite because I used to eat, have like a sugar craving.’’ PW2DM_20  ‘’Certainly, if they’ve had positive responses to it quite quickly, then obviously that reinforces that the medication is working well and therefore they’re encouraged to continue taking it. So yeah, certainly a positive improvement in their blood tests or in their numbers that they’re testing at home, or any – if there’s a medication that assists with weight reduction, then they’ve got some weight reduction quickly, then yeah, that’s certainly positive. It makes it easier to then encourage them to continue medications if they’re seeing that positive feedback.’’ Doctor_004 |
| **Intentions** | ‘’Yeah [ medications being a nightmare]. I wish I can stop doing this because I have to always carry medication around even if I'm traveling.’’ PW2DM_17  ‘’I might skip and I don’t really know whether it’s going to do anything if I just skipped.’’ PW2DM_17 | ‘’That is something I really wish I can try, but at the moment I don't have any evidence about herbal medicine to help me with..’’ PW2DM_17  ‘’Although sometimes I don’t take it very on time because I am stuck at work. However, I ensure I take it, and I won’t miss a dose.’’ PW2DM_6 |
| **Goals** |  | ‘’Good [diabetes medications] for my health, but, yeah, I want to live longer.’’ PW2DM_23 |
| **Memory, attention and decision processes** | ‘’I think if I’m really busy, I have an urgent thing to do, for example I have an appointment at the hospital then I might forget to take the medication, or I might do it when I come back.’’ PW2DM_17  ‘’Sometimes. I miss few times. Not a lot because like my iPhone to remind me. Sometimes if I don't have my iPhone close by in the evening and maybe I'm really tired after dinner, I'm sitting on the couch, watch a bit of tv, and I fell asleep.’’ PW2DM_18  ‘’I always struggled with taking medication regularly. I bought one of those small pill boxes but that didn't help because I would always forget about the pill box itself because I had to take two medications.’’ PW2DM_19  ‘’Yeah, I forgot sometimes so I don't really worry about that. As I said, I manage my diabetes mainly by diet because I'm really conscious about how much carbs I take each meal.’’ PW2DM_20    ‘’But if I miss the injection it doesn't matter with me because I just don't eat and that solves the problem.’’ PW2DM_12  ‘’The most common is just forgot it. They just forgot.’’ Doctor_003  ‘’Particularly with the Chinese, they won’t remember the drug name. It’s kind of like, this blue pill, this white pill, the big one, the small one. So, it’s really hard. So, you just have to have a photo of the brand’’ Doctor_003 | “t's become a regular part of my life, so I rarely forget. As Dr. advised, if I need to go out, I take my medication beforehand or carry it with me.’’ PW2DM_7  ‘’So I'm late for an hour I just do it an hour later. So I haven't really forget the whole dose, like the whole night or the whole morning completely. I always have to tell myself to remember, that's also a very hard. I mean it's also a very hard task that you have to keep doing.’’ PW2DM_17    “Oh, no. It's a routine for me. I get up first thing in the morning, I put all my tablets into one little container, I have injections. Takes me about 15 minutes." PW2DM_12  “I tick it off once I have the medication. Morning and night. On the paper. If you don’t do this, you don’t know if you’ve take it or not. This way, I can keep track of whether I have taken my medication or not.” PW2DM_13  “Mainly it's my - I reckon mine is my diet because if I'm eating less carbs and that I just - sometimes I forgot. I'm quite conscious about carbs. If I eat more carbs and I definitely would remember that.” PW2DM_20  “They be beneficial with using a Webster-pak so they can – if they are on a lot of regular medication sometimes they miss one or two for example, well a Webster-pak will be beneficial to them, to help them with their compliance issue …’’ Pharmacist_004 |
| **Environmental context and resources** | ‘’….the injection is always running out of stock. I have to visit different chemists to buy it. It's hard to keep it in stock.’’ PW2DM_6    ‘’No, I don't know what else I can do to change a machine or change a medication, the size of the medication. It's really up to the manufacturer to change it.’’ PW2DM_17  ‘’I think there is definitely a lack of information or resources in Mandarin, or other Chinese dialects, and that impacts on the ability to actually help their understanding of healthcare or specific medications. So, improving that would be a big resource.’’ Doctor_001  ‘’…so sometimes I actually - so occasionally when I go to interpreter, the Chinese interpreter is already booked, so I just let them interpret, and I find sometimes it's not very helpful. So anyway.’’ Doctor_005  ‘’Second thing is access to medicines, so with all the drug shortages it is challenging for patients [unclear] having consistent medication, so when things are out of stock, what do they do, that means they have to change something else, change to something else and they may not remember or they may not like it; no I like the old one better. That’s a negative thing. Some of these are out of our control.’’ Pharmacist_001  ‘’Yes, it's financially challenging. My pension doesn't cover all the costs, and I have to use my Chinese pension to buy medication here. I am not qualify for benefits here yet.’’ PW2DM_15  ‘’I don’t really speak or understand English, so language barriers has been an issues for me. I don’t know where to ask about the medication benefits.’’ PW2DM_15  ‘’The most popular one is still ongoing is Ozempic. Not sure if you have heard, but Ozempic is - can be used for weight loss. That is why there is a constant shortage of it. A lot of doctors have - or prescribers have changed patients over to other medications. But then that also means this other medication will go out of stock because everyone is using it too.’’ Pharmacist_002  ‘’Sometimes it can be, especially when pharmacies run out of stock. I need to go different pharmacy.’’ PW2DM_15  “Such materials aren’t readily available in general practices, and my GP does’t have any flyers or brochures when I asked. I think the information about dietary habits and diets are needed, especially considering the differences between Asian and Western eating habits, such as our focus on grains and soups. (…) We truly need it.” PW2DM_002 | “My pharmacist takes charge of my medication and delivers it to my place. I don’t need to pay either. He helps me restock every time.” PW2DM_14    ‘’Often, I find that there are medication errors. So, we might prescribe something, but they’ve misunderstood, and they’ve gone home and they’re taking it completely differently. So, often I like them to bring their medications physically, or at least take some photos. So, often if they’ve got a photo on their phone you can go through the tablets and identify them.’’ Doctor_004  ‘’I guess, it’s only applicable for one for the general medicine at the moment, where the patients, two days after being discharged a pharmacist will actually give them a call to touch base. We’re calling from Eastern Health, we want to just follow up with how you’re going, were there any questions with medicine.’’ Pharmacist_001  ‘’Having interpreters available. Not just Mandarin speaking interpreters but because there are some people coming from different backgrounds and different dialects, having that variety of dialects available is helpful as well.’’ Doctor_001  “A lot of our patients - Chinese background - it’s fine to have an interpreter, but after they go home, what do they read or see or watch? We probably need a formal, sort of a stream. Like a booklet or some sort of YouTube video that’s specific.” Pharmacist_005  “Having maybe a list of diabetes nurse educators who speak Mandarin or another Chinese dialect, around the area, would be helpful”. Doctor_001  “Australia here is doing quite a good job because we can still have multidisciplinary support of diabetes management" D2 "Currently with diabetes there is a lot more technology available, including continuous glucose monitoring. So, if the patient is utilising those resources, a lot of the time we have access to those results virtually as well." Doctor_002 |
| **Social influences** | ‘’I'm not comfortable taking medication in public. I'm not accustomed to sharing my health conditions with others; it's not necessary. They're not my doctors; they don't need to know.’’ PW2DM_5    ‘’I would not do it because I actually feel shame. I will feel shame when I do that in public so I always do it at home or somewhere people can't see me in the public.’’ PW2DM_17  ‘’I know some of my friends they take the herbal medicine as well but it’s not for everyone, especially some people from China they may do that but all other, you know the colleagues or friends I know, they will still taking the metformin or other [unclear] pharmacy medicines as well. I think the herbal may be better, but it applied to specific people depending on their culture and also the availability of the herbal medicine’’ PW2DM_24  ‘’I have some patients where they either have inquired or have tried either Chinese medications to help with their diabetes control, or they’ve asked around and they have friends who have tried diabetes – they’ve tried herbal medications for their health.’’ Doctor_001  “I guess what factors affect adherence, maybe sometimes they can be easily influenced by either family or friends, what they hear in their community. So, they might have some misconceptions or some negatives feeding back to that, you know, how medication might cause side effects or how medication might be associated with a bad outcome.” Doctor_004    ‘’So there’s two main types that they can use, yeah, but I know that some Chinese background patients they don’t actually believe in Western medicines, so that’s why they will rather do the Chinese medicine instead, which will be more like, to see a Chinese doctor and then they will prescribe the personalised medicine to themselves rather than seeing the Western doctor.’’ Pharmacist_004  “They have a heavy reliance and belief in Chinese medications and acupuncture and things like that. So, I tend to find that they always opt for those options first and put Western medications - or the medications that we use in hospital - as second. Pharmacist_005 | ‘’You need to trust the doctor. So, why you see the doctor? Because they are professional people.’’ PW2DM_4  ‘’I listen to doctors. I trust them. I take my medications on time.’’ PW2DM_5    ‘’Yes, I've told my friends about my medication and my situation. Some of my friends' brothers also have diabetes, and my friends are highly aware of it. They take preventive measures, such as exercising and monitoring their diet. Fortunately, they don't have diabetes.’’ PW2DM_13    ‘’So all my family and friends are fully aware of that and they just - everyone embraces the situation. So diabetes itself, tablet, no problem in our family.’’ PW2DM_18  ‘’They would help to remind me because this was really the first time that I had needed to take any regular medication and they would ask me, [Mr XXX], have you taken it? (…) I think that was the main way which they supported me.’’ PW2DM_19  ‘’Sometimes they will ask me, did you take your medication yet? Then if I couldn't be able to go and buy the medication they would get my prescription and help me to grab it.’’ PW2DM_22 |
| **Emotion** | ‘’I worry every day. I worried about my BGL rising when I go out to eat, I need to carry my medication everywhere, and I constantly monitor what to eat.’’ PW2DM_2  ‘’Because if I have to - because for type two diabetes once I have it I have it for life. So it’s really hard to always remember to take the medication. I don't just take one, I have to take two types, right. Injections are also very tedious to do, you have to do it every day, yeah, and I don't know how much it really helps with my diabetes.’’ PW2DM_17  ‘’I think my greatest concern was how long do I need to take it for because is it a medication that I will need to take for the short term or do I have to take it for the rest of my life.’’ PW2DM_19  ‘’Well, I was just quite worried about that because I don’t want to take insulin. I had the experience when I get pregnant and then it just feels like - I got a bruise on my tummy.’’ PW2DM_22    ‘’This is really too much. I have too much. I don't know when can I stop it [ diabetes medication].’’ PW2DM_25 | ’I feel like I take medication, I feel nothing wrong. Everything is normal, so that means I’m not worried about it.’’ PW2DM_4  ‘’It’s not a big deal if you miss one or two once in a while. I am not too concerned. But I always carry the medication with me when I go out. I feel I manage it well.’’ PW2DM_2  ‘’When you are a baby, you are getting everything, you getting your skill, you getting muscle and like you go to mature and after 35 or after 40 you come start to losing everything, losing your muscle, losing your strength, losing your health [laughs], taking medicine every day. That’s our life.’’ PW2DM_24 |
| **Behavioural regulation** | ‘’I always struggled with taking medication regularly. I bought one of those small pill boxes but that didn't help because I would always forget about the pill box itself because I had to take two medications. One wasn't - the other one wasn't for diabetes. It was for high blood pressure. It was [Candesartan] I think is the name. But no, it was a little bit awkward and difficult at times but there was no routine that helped me.’’ PW2DM_19 | “Low BGL often occur at night for me. I can feel the symptoms - dizziness, shakiness – and I will get up to eat something. I usually my BGL check at night. If it’s below 5, I will eat something before bed.” PW2DM_2  “But if I miss the injection it doesn't matter with me because I just don't eat and that solves the problem. To manage my own medication it's good. I know what to do.” PW2DM_12  “So yeah, certainly a positive improvement in their blood tests or in their numbers that they’re testing at home, or any – if there’s a medication that assists with weight reduction, then they’ve got some weight reduction quickly, then yeah, that’s certainly positive. It makes it easier to then encourage them to continue medications if they’re seeing that positive feedback” Doctor_004 |

PW2DM: People with type 2 diabetes mellitus; DNE: Diabetes Nurse Educator.
